# Do automatic push notifications improve patient flow in the emergency department? analysis of an ED in a large medical center in Israel

**DOI:** 10.1371/journal.pone.0258169

**Published:** 2021-10-07

**Authors:** Daniel Trotzky, Liron Posner, Jonathan Mosery, Aya Cohen, Shiran Avisar, Gal Pachys

**Affiliations:** 1 Department of Emergency Medicine, Shamir Medical Center (Assaf Harofeh Medical Center), Zerifin, Israel, affiliated with the Sackler Faculty of Medicine, Tel Aviv University, Tel-Aviv, Israel; 2 Division of Internal Medicine ‘D’, Shamir Medical Center (formerly Assaf Harofeh Medical Center), Zerifin, Israel, affiliated with the Sackler Faculty of Medicine, Tel Aviv University, Tel Aviv, Israel; 3 Shamir Medical Center (formerly Assaf Harofeh Medical Center), Zerifin, Israel, affiliated with the Sackler Faculty of Medicine, Tel Aviv University, Tel Aviv, Israel; Ohio State University Wexner Medical Center Department of Surgery, UNITED STATES

## Abstract

**Introduction:**

Congestion in emergency departments [ED] is a significant challenge worldwide. Any delay in the timely and immediate medical care provided in the ED can affect patient morbidity and mortality. Our research analyzed the use of an innovative platform to improve patient navigation in the ED, as well as provide updated information about their care. Our hope is that this can improve ED efficiency and improve overall patient care.

**Objective:**

The primary objective of our study was to determine whether the use of an automatic push notification system can shorten ‘length of stay’ (LOS) in the ED, improve patient flow, and decrease ED patient load.

**Methods:**

This was a prospective cohort study utilizing data extrapolated from the electronic medical records of 2972 patients who visited the walk-in ED of a large-scale central hospital in Israel from January 17, 2021 to March 15, 2021. During this period, the automatic push text notification system was activated on a week-on week-off basis. We compared data from our experimental group with the control group.

**Results:**

The results of this study indicate that the use of an automatic push notification system had a minimal impact on specific parameters of ED patient flow. Apart from a few significant reductions of specific timed-intervals during patients’ ED visit, the majority of results were not statistically significant.

**Conclusion:**

This study concluded that the anticipated benefits of a push text notification system in the ED do not, at this stage, justify the system’s additional cost. We recommend a follow-up study to further investigate other possible benefits.

## Introduction

Congestion and overcrowding in emergency departments [ED] is a significant challenge worldwide [1]. The growing number of patients, increased task load, along with a greater variety of diagnostic and therapeutic tools at clinicians’ disposal has contributed to ED congestion [2]. The bottleneck created at this critical gateway to the greater hospital network has increased the length of stay (LOS) in the ED and negatively affected patient care. As timely, efficient and life-saving care is the mainstay of ED work, any delay in care directly affects patient morbidity and mortality [3]. Inefficient patient care as well as rising financial costs have spurred national governments to address the problem and optimize efficiency through improved patient flow and reduced waiting times [4].

Research studies and programs aimed at decreasing emergency department wait times have been reported in the literature. The "Australian Emergency Nursing Journal" published a case-control trial that investigated the role of a "navigator nurse" in the ED to facilitate efficient patient movement. The results of the trial demonstrated that ED LOS was significantly reduced on days that the navigator was operating as well as the time until the patient was seen by a treating physician [4].

In 2015, Israel’s ministry of health [MOH] set out to better understand and analyze the factors contributing to and the repercussions of increased ED congestion. Various parameters of patient experience in the ED work were evaluated including wait times in the ED, the time to first referral, time to decision, and overall length of stay [LOS] in the ED. Israel’s MOH then initiated a program titled the "incentive model" which offered incentives to healthcare institutions which demonstrated measurable improvement in the overall ‘ED experience’. A central pillar of this ‘incentive model’ was the implementation of a patient management software called Q-Flow [5]. The Q-Flow system is a patient management software designed for use in walk-in clinics of the ED. The system tracks a patient’s progress and manages waiting times at various physician offices and diagnostic tests for optimal efficiency in the ED [6].

Israel’s fourth largest government hospital, Shamir—Assaf Harofeh Medical Center, successfully implemented the use of the Q-Flow system in the ED walk-in clinic as part of the national initiative in 2015. Shamir Medical Center is an 848-bed academic medical facility that provides care for over 1 million residents of Israel’s central region. The emergency medicine department is the fourth largest in Israel, treating about 160,000 patients each year. The ED is divided into the medicine, surgery, pediatrics and obstetrics-gynecology services. Approximately half of the patients presenting to the ED are triaged and cared for in the walk-in or ambulatory ED.

The ambulatory ED encompasses many unique stations, each with their own characteristics, such as a triage, examination, imaging and physicians’ room. Patients must navigate between the various stations and move between multiple waiting rooms independently. As a result, patients often mistakenly wait in incorrect locations or are delayed in arriving at their next station. Occasionally patients are directed to the wrong location, and as a result, either arrive late or miss their allocated time slot.

Our study investigated an innovative platform utilizing automated push notifications used in conjunction with the existing Q-Flow software. The automated text message push notification system platform draws information from the Q-flow patient management software and delivers automatic notifications to patients. Our hope was that this tool would improve overall efficiency in the ED and reduce unnecessary patient friction through delivery of information to patients and accurate instructions as they navigate through the ED. Digital automatic notifications can deliver timely communication, as well as reduce workload by limiting human interaction for non-urgent issues, allowing providers to focus on more urgent patient care [7].

At Shamir Medical Center’s ED, patients were automatically enrolled for push notifications upon their registration at the ED. The notifications accompanied patients throughout their visit. Short notifications via text messages were sent in real time to their phone confirming completion of a task at a specific station, such as imaging or laboratory test, and directing them to their next station, including its time and location. The primary objective of our study was to determine whether the use of automatic push notifications as a navigation tool can shorten ‘length of stay’ (LOS) in the ED, improve patient flow, and decrease ED load. This information can assist ED directors in understanding the cost-benefit analysis of potential tools to improve ED efficiency. Few studies have been published analyzing the use of web-based patient-focused applications in the ED.

The primary objective of our study was to determine whether the use of an automatic push notification system can shorten ‘length of stay’ (LOS) in the ED, improve patient flow, and decrease patient load. The results of this study did not support our hypothesis. Nonetheless, we believe a further study is necessary to determine if the system may have contributed in other unique ways. In particular, the system may provide ED directors with an important automatic tool to improve patient flow and overall patient satisfaction by providing a more transparent and clearer road map for patients as they navigate the ED.

## Materials and methods

### Study design and settings

This was a prospective study utilizing data extrapolated from electronic medical records of patients who visited the walk-in ED of a large-scale hospital in central Israel from January 17^th^ 2021 to March 15^th^ 2021. The Institutional ethics committee of Shamir Medical Center approved the study and waived the requirement for written informed consent, reference 0391–20.

### Participants

The final study sample consisted of 2972 patients, who attended the walk-in clinic of the ED between January 17^th^—March 15^th^, 2021 and met our inclusion criteria. Initially, of all the visitors that were admitted to the ED during the mentioned period [n = 16,987], we extracted only the files of those who were directed to the walk-in clinic [n = 4163]. We later excluded visitors under the age of 18 [n = 328], visitors for which the "Time to Triage" wasn’t documented [n = 250], or was documented as 0, 1 and over 90 minutes [n = 77], visitors who were immediately discharged or admitted to hospital at time of admission to ED [n = 23] and visitors who’s documented "total LOS" in the ED was 0, 4 and over 250 minutes [n = 45].

**Fig 1:**
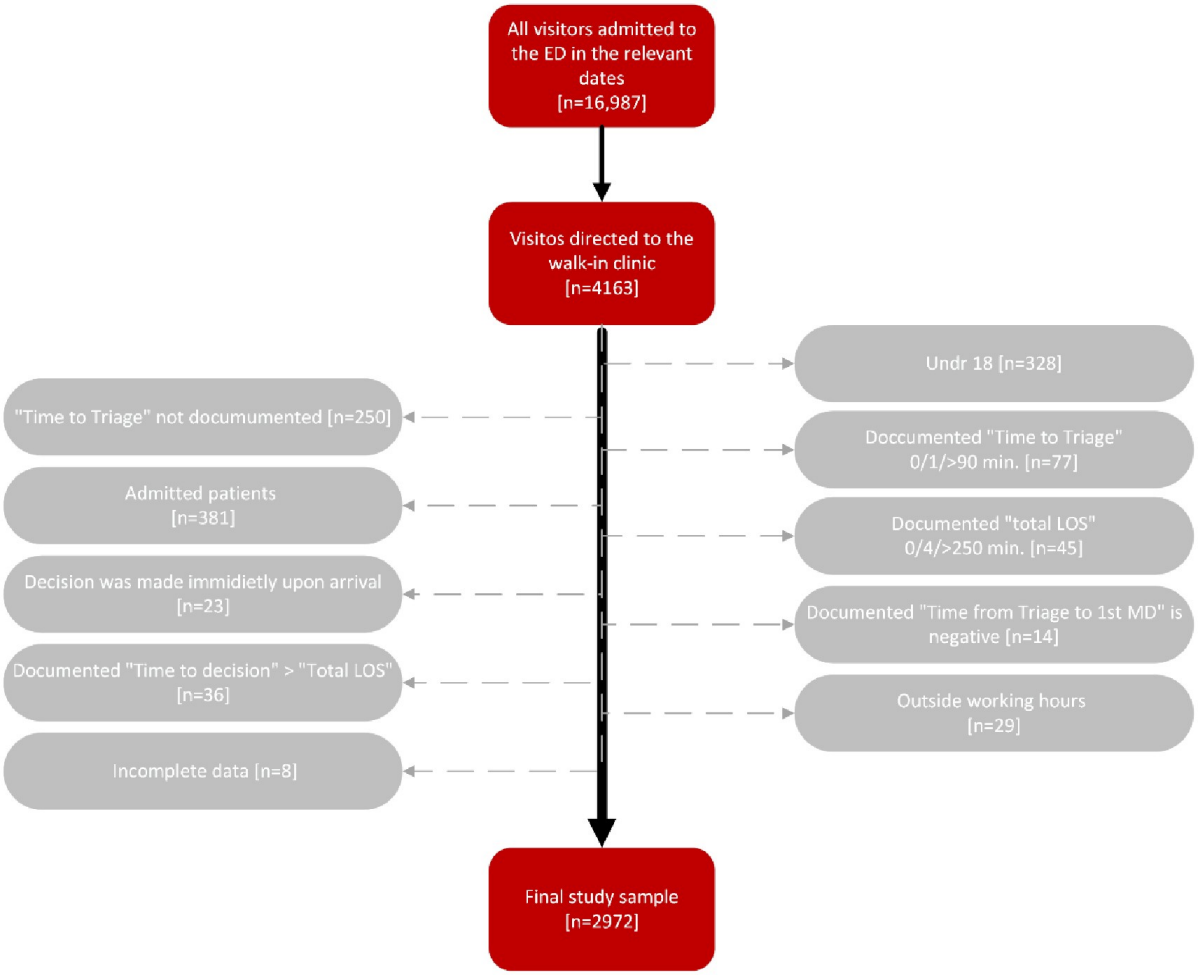


Patients who were admitted to the hospital were excluded from the study, as push notifications from the SMS system had no impact on the time from ‘decision to admit’ to actual hospitalization [n = 381]. In addition, in order to ensure that the "Time to first MD" will be longer than the "Time to Triage", we excluded visitors for which the documented "Time from Triage to first^t^ MD" was negative [n = 14]. We also excluded those patients for which the documented "Time to decision" was longer than the documented "Total LOS"[n = 36]. Lastly, we excluded visitors who were documented to have attended the walk-in clinic outside its working hours [n = 29] as well as patients with incomplete data in their medical records [n = 8] (Fig 1).

### Procedure

Between January 17^th^ and March 15^th^ 2021, the automatic push text notification system was activated, intermittently, on a week-on week-off basis. Every patient who attended the walk-in clinic during this period was recruited to the study upon arrival. Upon registration at the main desk, receptionists updated the patient’s cell number, assuring that the SMS would be delivered to the intended recipient. At the end of the test trial period, we extracted operational data, retrospectively, using the Q-flow system. The extracted data included the "Time to triage", "Time to first MD", " Time from Triage to first MD”, "Time to decision", and overall LOS in the ED. We then compared the results from our experimental group which consisted of patients who visited the clinic on the weeks that the system was activated, with the results from the control group of patients who visited the clinic on the weeks the system was not activated.

To assess the operational efficiency of the text system, we compared its contribution, calculated by a number of different variables, at different ‘workload periods’ [see Table 1]. We divided ‘workload periods’ in the walk-in ED according to the number of new admissions per hour. High workload was considered as >200 admission/hour, moderate workload as 150–200 admissions/hour and light workload as <150 admissions/hour.

**Table 1:** Workload periods by admissions per hour.

Workload	Level	Admissions Per Hour
Load 1	High	> 200
Load 2	Moderate	150–200
Load 3	Light	< 150

The ED at Shamir Medical Center is divided into three services: internal medicine, which we classified as ‘Service 1’, orthopedics as ‘Service 2’, and surgery/minor trauma as ‘Service 3’.

### Variables

The primary outcome of the study is the ‘Total LOS’, measured from arrival at the ED to discharge [see Table 2]. Secondary outcomes included ‘Time to Triage’ [measured from arrival to 1^st^ encounter with triage nurse], ‘Time to first MD’ [measured from arrival to first encounter with a treating physician], ‘Time from Triage to first ^t^ MD’ [measured from first encounter with triage nurse to first encounter with treating physician] and ‘Time to decision’ [measured from arrival to decision of discharge/admission] [see Table 3].

**Table 2:** Primary outcome.

Measured variable	Time measured
Total LOS	Arrival 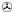 discharge

**Table 3:** Secondary outcomes.

Measured variable	Time measured
Time to triage	arrival 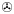 first encounter with triage nurse
Time to first MD	arrival 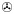 first encounter with a treating physician
Time from Triage to first MD	first encounter with triage nurse 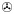 first encounter with treating physician
Time to decision	arrival 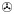 decision of discharge/admission

### Data sources

Information was extracted from a single database system called "Namer" [SAP (Systems, Applications, and Products in Data Processing) Israel ltd., Ra’anana, Israel], an electronic health record system, based on the SAP platform. The “Namer” is used in 10 government hospitals in Israel including Shamir Medical Center. It records nearly all of the patient’s visit data including identifying details, administrative information, assessment, test results, treatments, decisions and physician.

### Data management and statistical analysis

Categorical variables were reported as frequency and percentage. Continuous variables were evaluated from normal distribution using histogram, and reported as median and interquartile range [IQR]. Chi-Square Test was used to compare categorical variables, while Mann—Whitney Test was used to compare continuous and ordinal variables. All statistical tests were two—sided and P < 0.05 was considered as statistically significant. SPSS software [IBM SPSS statistics for windows, version 24, IBM corp., Armonk, NY, USA, 2016] was used for all statistical analysis.

## Results

Raw data were retrieved from the "Namer" database for all patients that presented to the ED from January 17, 2021 to March 15, 2021. After data cleaning, 2972 patient files were analyzed. The mean age of the sample was 42.4 years [SD 18.9] and the majority was male [53.5%, n = 1590]. No statistical difference was found in any socio-demographic characteristics. In terms of presentation numbers, the busiest hour of the day was 11:00 am [9.3%, n = 276] and the quietest was 23:00 pm [1.3%, n = 40]. Workload 1 was the busiest one [70.1%, n = 2083] and Workload 3 was the quietest [11.4%, n = 338]. Almost half of all patients were in triage category 3 [48.1%, n = 1429]. Sample characteristics were compared between the days the SMS system was activated and the days it was not. [see Table 4].

**Table 4:** Sample characteristics.

*sample characteristics*		*SMS system*		
		Off	On	Total
***Total n [%]***		1505 [50.6]	1467 [49.4]	2972 [100]
***Mean age [SD]***		42.27 ±18.7	42.51 ± 19.2	42 ± 18.9
***Gender n [%]***	M	806 [50.7]	784 [49.3]	1590 [53.5]
	F	699 [50.6]	683 [49.4]	1382 [46.5]
***Load n [%]***	1	1040 [49.9]	1043 [50.1]	2083 [70.1]
	2	274 [49.7]	277 [50.3]	551 [18.5]
	3	191 [56.5]	147 [43.4]	338 [11.4]
***Triage category n [%]***	2	28 [60.9]	18 [39.1]	46 [1.5]
	3	720 [50.4]	709 [49.6]	1429 [48.1]
	4	562 [52.5]	508 [47.5]	1070 [36.0]
	5	193 [45.4]	232 [54.6]	425 [14.3]

### Time-based performance indicators

Differences in time-based performance indicators were analyzed and are shown in Table 5. On the days that the SMS system was activated, reduced ED time intervals were documented at all the stations, although none of the parameters were of statistical significance except for the mean "time to first MD" which was significantly reduced by around 3.5 min. with a p value of 0.004.

**Table 5:** Time-based performance indicators.

*Time intervals [min*.*]*	*SMS system mean*, *median*, *n*		*Mean difference [95% CI]*	*Significance [p]*
	Off	On		
***Time to triage***	21.57, 17, 1505	20.06, 17, 1467	1.51	0.53
***Time to 1***^***st***^ ***MD***	65.56, 55, 1505	61.97, 56, 1467	3.59	0.004
***Time from triage to 1***^***st***^ ***MD***	44.02, 34, 1505	41.91, 34, 1467	2.11	0.095
***Time to decision***	173.64, 143, 1505	166.24, 143, 1467	7.4	0.299
***Total LOS***	204.54, 172, 1505	198.54, 173, 1467	6	0.482

### Comparison between workloads

In Table 6 we compared the time intervals of the different stations between 3 different workloads. A significant time reduction was observed in load 2 with reduced mean "time to first MD" by around 7.5 min. [p = 0.03]. another significant reduction was seen in load 3 with reduction of about 4.8 min. in the mean "time to triage" [p = 0.003].

**Table 6:** Comparison between workloads.

	*Time intervals [min*.*]*	*SMS system mean*, *median*, *n*		*Mean difference [95% CI]*	*Significance [p]*
		Off	On		
***Load 1***	**Time to triage**	22.48, 17, 1040	21.77, 17, 1043	0.71	0.579
	**Time to first MD**	66.35, 55, 1040	64.12, 56, 1043	2.23	0.055
	**Time from triage to first MD**	43.9, 34, 1040	42.36, 34, 1043	1.54	0.128
	**Time to decision**	185.84, 143, 1040	177.12, 143, 1043	8.72	0.266
	**Total LOS**	216.1, 172, 1040	209.7, 173, 1043	6.4	0.552
***Load 2***	**Time to triage**	20.24, 16, 274	17, 17, 277	3.24	0.083
	**Time to 1**^**st**^ **MD**	65.57, 56, 274	58.01, 56, 277	7.56	0.03
	**Time from triage to first MD**	45.35, 35, 274	41.02, 34, 277	4.33	0.353
	**Time to decision**	158.86, 144, 274	153.36, 143, 277	5.5	0.709
	**Total LOS**	190.14, 174, 274	180.65, 173, 277	9.49	0.367
***Load 3***	**Time to triage**	18.52, 17, 191	13.74, 17, 147	4.78	0.003
	**Time to first MD**	61.25, 56, 191	54.16, 56, 147	7.09	0.188
	**Time from triage to first MD**	42.74, 35, 191	40.37, 34, 147	2.37	0.992
	**Time to decision**	128.36, 143, 191	113.25, 144, 147	15.11	0.295
	**Total LOS**	162.31, 173, 191	153.02, 173, 147	9.29	0.561

*Load 1 > 200 admissions/hour | Load 2 150–200 admissions/hour | Load 3 < 150 admissions/hour.

### Comparison between services

Table 7 compares the time intervals of the different stations between the three services, each subdivided into three workloads. Service 1 refers to Internal medicine, Service 2 to orthopedics and Service 3 to surgery/minor trauma. A significant reduction in time was only notable in the orthopedic patients [Service 2], in the "time to first MD" during load 1 [mean difference 5.47 minutes, p = 0.022] and in the "time to triage" in load 2 [mean difference 4.08 minutes, p = 0.036] & load 3 [mean difference 5.73 minutes, p = 0.004].

**Table 7:** Comparison between services.

		*Time intervals [min*.*]*	*SMS system mean*, *median*, *n*		*Mean difference [95% CI]*	*Significance [p]*
			Off	On		
***Service 1***	***Load 1***	**Time to triage**	22.85, 17, 389	22.64, 17, 384	0.21	0.957
		**Time to first MD**	62.63, 56, 389	61.66, 56, 384	0.97	0.245
		**Time from triage to first MD**	39.78, 34, 389	39.03, 34, 384	0.75	0.091
		**Time to decision**	221.33, 144, 389	202.76, 143, 384	18.57	0.067
		**Total LOS**	244.15, 173, 389	232.16, 173, 384	11.99	0.288
	**Load 2**	**Time to triage**	22.04, 17, 107	19.09, 17, 112	2.95	0.788
		**Time to first MD**	62.39, 56, 107	58.53, 56, 112	3.86	0.803
		**Time from triage to first MD**	40.33, 34, 107	39.48, 34, 112	0.85	0.508
		**Time to decision**	177.12, 145, 107	175.99, 143, 112	1.13	0.685
		**Total LOS**	202.32, 173, 107	194.58, 173, 112	7.74	0.397
	**Load 3**	**Time to triage**	16, 16, 37	14.23, 17, 31	1.77	1
		**Time to first MD**	60.7, 55, 37	53.48, 56, 31	7.22	0.735
		**Time from triage to first MD**	44.68, 35, 37	39.26, 34, 31	5.42	0.522
		**Time to decision**	225.95, 144, 37	183.9, 144, 31	42.05	0.614
		**Total LOS**	244.57, 173, 37	208.71, 173, 31	35.86	0.946
***Service 2***	**Load 1**	**Time to triage**	21.55, 17, 389	20.11, 17, 378	1.44	0.340
		**Time to first MD**	65.77, 55, 389	60.30, 56, 378	5.47	0.022
		**Time from triage to first MD**	44.28, 34, 389	40.18, 34, 378	4.1	0.160
		**Time to decision**	122.11, 143, 389	112.47, 143, 378	9.64	0.289
		**Total LOS**	164.97, 172, 389	155.41, 173, 378	9.56	0.561
	**Load 2**	**Time to triage**	19.17, 17, 102	15.09, 17, 96	4.08	0.036
		**Time to first MD**	67.25, 55, 102	58.72, 56, 96	8.53	0.086
		**Time from triage to first MD**	48.18, 34, 102	43.66, 34, 96	4.52	0.281
		**Time to decision**	109.52, 143, 102	105.60, 143, 96	3.92	0.612
		**Total LOS**	154.63, 172, 102	143.29, 173, 96		0.664
	**Load 3**	**Time to triage**	19.90, 17, 131	14.17, 17, 103	5.73	0.004
		**Time to first MD**	60.41, 56, 131	53.54, 56, 103	6.87	0.118
		**Time from triage to first MD**	40.56, 35, 131	39.32, 34, 103	1.24	0.731
		**Time to decision**	94.93, 143, 131	86.24, 143, 103	8.69	0.262
		**Total LOS**	136.79, 173, 131	134.05, 173, 103	2.74	0.318
***Service 3***	**Load 1**	**Time to triage**	23.33, 17, 262	22.81, 17, 281	0.52	0.952
		**Time to first MD**	72.75, 56, 262	72.63, 56, 281	0.12	0.999
		**Time from triage to first MD**	49.44, 34, 262	49.83, 34, 281	-0.39	0.802
		**Time to decision**	227.78, 145, 262	229.06, 143, 281	-1.28	0.724
		**Total LOS**	250.34, 173, 262	252.05, 173, 281	-1.71	0.792
	**Load 2**	**Time to triage**	18.95, 17, 65	16.25, 17, 69	2.7	0.324
		**Time to first MD**	68.14, 56, 65	56.20, 56, 69	11.94	0.072
		**Time from triage to first MD**	49.20, 35, 65	39.86, 34, 69	9.35	0.191
		**Time to decision**	206.25, 145, 65	183.09, 143, 69	23.16	0.384
		**Total LOS**	225.83, 173, 65	210.03, 172, 69	15.8	0.465
	**Load 3**	**Time to triage**	14.70, 16, 23	9.23, 17, 13	5.47	0.060
		**Time to first MD**	66.87, 55, 23	60.69, 56, 13	6.18	0.818
		**Time from triage to first MD**	52.09, 35, 23	51.38, 34, 13	0.71	0.767
		**Time to decision**	161.74, 144, 23	158.77, 143, 13	2.97	0.830
		**Total LOS**	175.35, 174, 23	170.54, 172, 13	4.81	0.693

*Service 1 = internal medicine | Service 2 = orthopedics | Service 3 = surgery/minor trauma.

*Load 1 > 200 admissions/h | Load 2 150–200 admissions/h | Load 3 < 150 admissions/h.

## Discussion

Our main objective was to determine whether the use of an innovative push notification system would decrease ED length-of-stay (LOS). Unfortunately, in spite of our optimism, our hypothesis proved to be false. We did not observe a significant reduction in the mean "total length-of-stay (LOS)" in the experimental group of patients.

We did observe significant time reductions in ‘Time to Triage’, ‘Time to first MD’, Time from ‘Triage to first MD’ and ‘Time to Decision’. In the orthopedic service, which is located at the furthest site of the walk-in clinic, we observed, on average, a 5-minute reduction in the mean ‘Time to first MD’ and mean ‘Time to triage’. We also observed a significant reduction in the lighter workloads [<200 admissions/hour], where a reduction of 7.5 minutes to first MD was noted during load 2, and a 4.7-minute reduction of the mean time to triage in load 3. In addition, the mean ‘Time to first MD’ was also reduced by 3.6 minutes, regardless of service or load. Apart from the minor time reductions mentioned above, no other statistically significant differences were observed and our primary hypothesis of shortened ED LOS wasn’t supported.

Although we did not find any significant benefit from the use of the push text notification system in this study, we do believe that it had an overall positive effect on patients’ experience, and we therefore recommend a follow-up study to evaluate patient satisfaction from the presence of this navigation system. Furthermore, automated messages served as an additional confirmation to instructions provided by staff and reduced errors in the event that a patient was given erroneous or inaccurate instructions.

Few studies have analyzed the use of web-based applications that provide automatic direct patient updates and notifications during an ED visit with the purpose improving patient flow and ED efficiency [8]. Furthermore, we have not found any material that specifically analyzes a text message push notification system that sends directions to ED patients for their next station or assignment in the ED. We believe there is potential in further exploring this and other similar technological tools.

This article is important as it demonstrates that the benefits of using an automated push text notification system do not, at this stage, appear to justify its costs. The use of this system should therefore be reconsidered.

## Conclusion

To our knowledge, this is the first study of its kind to analyze the use and benefits of an automated push notification system in a walk-in clinic of an ED. The results from this study indicate that the use of an SMS push text notification system in the walk-in clinic of the Shamir Medical Center had a minimal positive impact on specific parameters of ED patient flow. However, apart from a few statistically significant reductions of observed timed-intervals during patients’ ED visit, the majority of results were not statistically significant, the majority of the results were not significant.

Nonetheless, we believe that this innovative platform did have an impact on the overall patient experience in the ED and we therefore recommend a follow-up study to further investigate this hypothesis. Small improvements, in both patient satisfaction and improved efficiency in the ED, can have a significant impact on mortality and morbidity in the ED.

## References

[1] McKenna P, Heslin SM, Viccellio P, Mallon WK, Hernandez C, Morley EJ. Emergency department and hospital crowding: Causes, consequences, and cures. *Clin Exp Emerg Med*. 2019;6(3):189–195. doi: 10.15441/ceem.18.022 31295991 PMC6774012

[2] Lindner G, Woitok BK. Emergency department overcrowding: Analysis and strategies to manage an international phenomenon. *Wien Klin Wochenschr*. Published online January 13, 2020:1–5. doi: 10.1007/s00508-019-01596-7 31932966

[3] Jessup M, Fulbrook P, Kinnear FB. Multidisciplinary evaluation of an emergency department nurse navigator role: A mixed methods study. *Aust Crit Care*. 2018;31(5):303–310. doi: 10.1016/j.aucc.2017.08.006 28941792

[4] Fulbrook P, Jessup M, Kinnear F. Implementation and evaluation of a ‘Navigator’ role to improve emergency department throughput. *Australas Emerg Nurs J*. 2017;20(3):114–121. doi: 10.1016/j.aenj.2017.05.004 28624270

[5] Ministry of Health Emergency Department Incentive Model 2017. health.gov.il. (2018). https://www.health.gov.il/NewsAndEvents/SpokemanMesseges/Pages/30012018_1.aspx

[6] Patient Management Software, Q-nomy Health. (2017) https://www.qnomyhealth.com/q-flow-platform

[7] Agarwal AK, Sangal RB, Hahn L, et al. Digital Care Updates in the Emergency Department: A Feasibility Study. Griffey R, ed. *Acad Emerg Med*. 2020;27(3):236–239. doi: 10.1111/acem.13905 31837070

[8] Salmasian H, Landman AB, Morris C. An electronic notification system for improving patient flow in the emergency department 1 Brigham and Women ‘ s Hospital and Harvard Medical School, Boston, MA.: 242–247. PMC6568086 31258976

